# Comparison of reference systems in the assessment of age-related serum immunoglobulin levels in pediatric patients

**DOI:** 10.3906/sag-1805-220

**Published:** 2019-02-11

**Authors:** İlknur KÜLHAŞ ÇELİK, Ersoy CİVELEK, Ayşe METİN, Tayfur GİNİŞ, Müge TOYRAN, Emine DİBEK MISIRLIOĞLU, Can Naci KOCABAŞ

**Affiliations:** 1 Ankara Child Health and Diseases Hematology Oncology Training and Research Hospital,Division of Pediatric Allergy and Immunology, University of Health Sciences, Ankara Turkey; 2 Division of Pediatric Allergy and Immunology, Department of Children’s Health and Diseases,Faculty of Medicine, Muğla Sıtkı Koçman University, Muğla Turkey

**Keywords:** Children, reference system, serum immunoglobulin levels

## Abstract

**Background/aim:**

Ig level assessment is frequently used in the diagnosis and follow-up of immunodeficiency, as well as in studies investigating the prevalence of low serum Ig level in specific diseases.**Material and methods:** Patients who underwent Ig testing in the inpatient and outpatient clinics of our hospital in the years 2010–2016 were included. The Ig levels of the patients were assessed separately according to two reference systems commonly used in Turkey and another reference system used in the USA.

**Results:**

A total of 20,138 patients (57.6% male) were included in the study. The median age of the patients was 55.7 months (interquartile range: 23.1–96.7). According to the reference intervals determined by Tezcan et al., 30.6% of the patients were deficient in one or more Ig values. This rate was 4 times higher than those based on the reference intervals determined by Aksu et al. (7.7%) and those in the *Nelson Textbook of Pediatrics* (6.8%). We also determined that the frequency of low Ig levels with three reference systems.**Conclusion**: In this study, we found that the rates of low Ig level in a group of pediatric patients differed significantly when evaluated using three different reference systems for age-related serum Ig levels

## 1. Introduction

Primary immunodeficiencies are inherited immune system disorders that cause increased frequency and severity of infections and predisposition to autoimmune disease and malignancies. They occur at a rate of 1:2000 at live births (1). The most common group of primary immunodeficiencies are the primary antibody deficiencies, which cause disorders in antibody production and function (2,3). Evaluation of patients with antibody deficiency should include B-lymphocyte count, serum total immunoglobulin (Ig) level measurement, and assessment of specific antibodies against protein and polysaccharide antigens (4).

Maturation of the immune system and Ig levels begins in the neonatal period and continues into childhood. In healthy newborns, the serum IgG level matches that of the mother due to placental transfer in the last trimester; however, this level falls around 4–6 months after birth, then gradually increases again to adult level by the age of 5 or 6 years. IgA and IgM levels are very low in the neonatal period due to lack of encounters with foreign antigens. IgM reaches adult levels at about one year of age, while IgA reaches adult levels during adolescence. These processes result in age-related differences in Ig levels (4). In addition to age, genetic and environmental factors (frequent and/or prolonged antigenic stimulation) also affect Ig levels. Therefore, age-related reference intervals of healthy individuals in the population must be used when evaluating a patient’s Ig levels (5).

An important problem in the determination of reference intervals is that these values vary by region and source population, as well as with laboratory and technical conditions. The International Federation of Clinical Chemistry (IFCC) recommends that each laboratory determine its own values (6). 

Immunological methods such as radial immunodiffusion, enzyme-linked immunosorbent assay (ELISA), and immunonephelometry have been used to measure IgG and IgG subgroups. The sensitivity of the method also influences reference values. Furthermore, differences in calibration between methods pose a major challenge in the comparison of reference intervals. For this reason, calibration based on an international reference material is recommended (7). 

The aim of the present study was to classify the results of Ig level measurements requested in the pediatric units of our hospital for various reasons as low or normal for the patient’s age using two different reference systems used in Turkey (5,8) and a reference system used in the US (9) and to determine whether the three systems yielded different proportions of patients evaluated as having low and normal Ig levels.

## 2. Materials and methods 

### 2.1. Patient population

Patients who underwent Ig testing in the outpatient and inpatient units of the Ankara Children’s Health and Diseases Hematology Oncology Training and Research Hospital of the Department of Health Sciences University between January 2010 and January 2016 were included. All patient data were obtained from their medical records and from the hospital database. The study was approved by the local ethics committee (2018-046).

### 2.2. Measurement of serum immunoglobulin levels

Immunoglobulin measurements were performed with an IMMAGE 800 Immunochemistry System using the nephelometric method and values were expressed as mg/dL.

### 2.3. Classification of low/normal immunoglobulin levels according to age 

The patients’ Ig values were classified as low or normal according to the relevant age-related reference intervals for each Ig value. For these reference intervals, we used two reference systems (5,8) based on age-related normal Ig ranges determined in Turkish children, and one reference system used in the United States (9).

The percentage of patients with low and normal Ig levels in each age group was determined for each of the three references.

### 2.4. Statistical analysis

Statistical analysis of the data was done with the SPSS for Windows version 22 software package. Categorical variables were expressed as number and percentage, continuous variables as minimum–maximum, mean ± standard deviation, and median. Chi-square test was used to compare continuous variables between two independent groups. A P-value <0.05 was considered statistically significant.

## 3. Results

A total of 20,138 patients (57.6% male) were included in the study. The median age of the patients was 55.7 months (interquartile range: 23.1–96.7 months). 

Evaluation of the patients’ Ig values according to the reference interval determined in Turkey by turbidimetry, 6174 (30.6%) of the 20,138 patients were found to have one or more Ig values lower than the age-related reference interval. Of these 6174 patients, deficiencies were detected in IgA-only in 35.4% (n = 2.186), IgG-only in 25.8% (n = 1.533), IgM-only in 10.6% (n = 657), IgA and IgG in 14.4% (n = 890), IgA and IgM in 6.2% (n = 383), and IgM and IgG in 2.9% (n = 178). Low levels of all three Ig types (G, A, M) were detected in 5.6% (n = 347) of this group.

Using the other Turkish reference intervals determined by immunonephelometry, 1557 (7.7%) of the 20,138 patients were found to have one or more Ig values lower than the age-related reference intervals. Of these patients, deficiencies were detected in IgA-only in 56% (n = 873), IgG-only in 20.5% (n = 319), IgM-only in 8.9% (n = 139), IgA and IgG in 8.7% (n = 136), IgG and IgM in 2.2% (n = 34), and IgA and IgM in 2.1% (n = 33). Low levels of all three Ig types were detected in 1.5% (n = 23) of this group.

Using the reference intervals determined in the US by immunonephelometry, 1368 (6.8%) of the 20,138 patients had one or more Ig levels below the age-related reference interval. Of these, the deficiencies were detected in IgA-only in 48.9% (n = 670), IgM-only in 32.7% (n=448), IgG-only in 8.2% (n = 113), IgA and IgM in 4% (n = 54), IgG and IgM in 2.7% (n = 37), and IgA and IgG in 1.5% (n = 21). Low levels of all Ig types were detected in 1.8% (n = 25) of this group.

**Figure 1:**
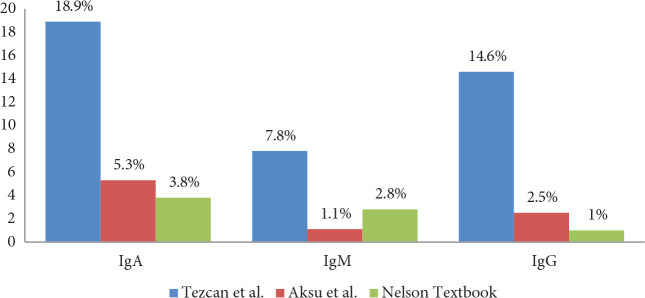
The rates of low Ig levels with the three reference systems.

The rates of low Ig levels with the three reference systems are shown in Figure 1.

**Figure 2:**
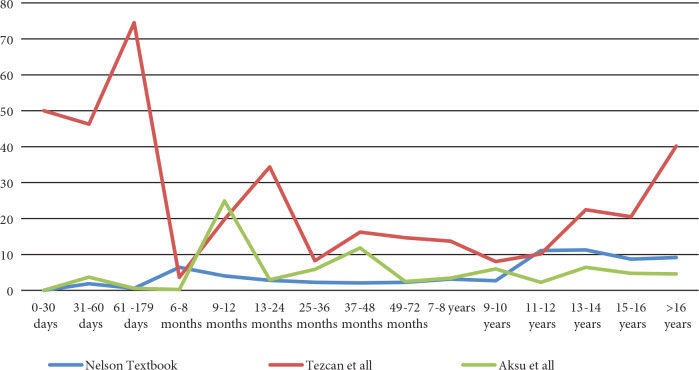
The frequency of low IgA levels with the three reference systems according to age.

**Figure 3:**
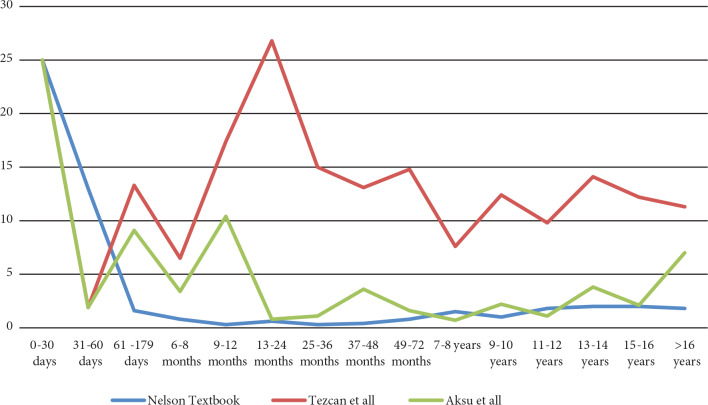
The frequency of low IgG levels with the three reference systems according to age.

**Figure 4:**
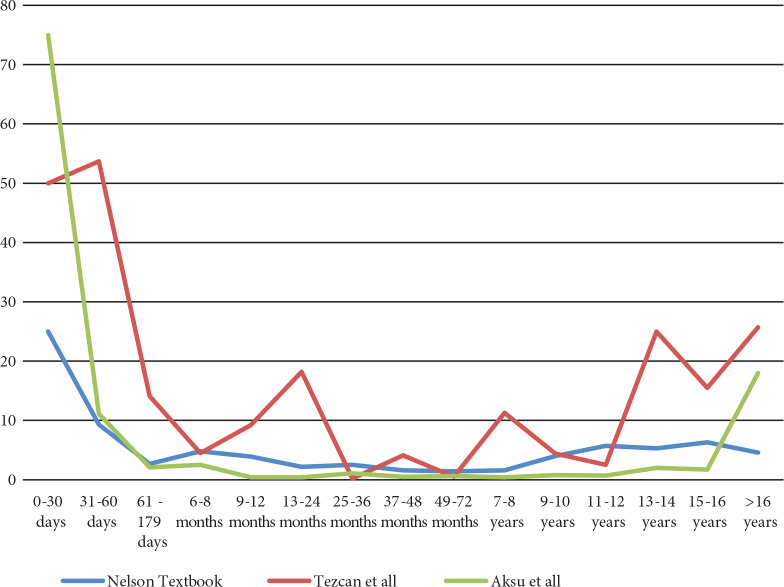
The frequency of low IgM levels with the three reference systems according to age.

We also determined that the frequency of low Ig levels with the three reference systems differed according to age (Figures 2–4). We found that there is even more difference between reference systems in patients under one year of age.

The 20,138 study patients were separated into two age groups (<4 years and >4 years) and evaluated separately because of its importance in terms of transient hypogammaglobulinemia of infancy. In total, 8973 patients (44.6%) were under 4 years of age and 11,165 patients (55.4%) were over 4 years of age. The proportions of low Ig levels in patients under and over 4 years of age based on the three reference systems are presented in Table.

**Table 1:** Patients in the <4 year and >=4 year age groups evaluated as having low immunoglobulin levels according to three different reference systems.

		Patients with low Ig level, n (%)	
Immunoglobulin		Patient age <4 years	Patient age >=4 years
IgA	Tezcan et al.	2139 (23.8%)	1667 (14.9%)
	Aksu et al.	672 (7.5%)	393 (3.3%)
	Nelson	255 (2.8%)	515 (4.6%)
IgG	Tezcan et al.	1562 (17.4%)	1386 (12.4%)
	Aksu et al.	310 (3.59%)	202 (1.8%)
	Nelson	58 (0.6%)	138 (1.2%)
IgM	Tezcan et al.	816 (9.1%)	749 (6.7%)
	Aksu et al.	91 (1%)	138 (1.2%)
	Nelson	242 (2.7%)	322 (2.9%)

## 4. Discussion

Obtaining a thorough history, especially a detailed family history, is as crucial in the diagnosis of immune deficiencies as laboratory studies. Accordingly, detecting low Ig levels alone is not sufficient for a diagnosis of primary immunodeficiency. However, serum Ig levels used in the diagnosis and follow-up of various immunological diseases are useful indicators of B and T cell development and function (10). Ig level assessment is frequently used to diagnose and monitor immunodeficiency, as well as in studies investigating the prevalence of low Ig level in specific disease groups, particularly allergic conditions (11–13). The results of laboratory analyses play an important role in clinicians’ decisions regarding diagnosis and follow-up; therefore, the reference intervals for laboratory results should be definite and specific to the patient population (14).

When we evaluated two reference systems used in Turkey and another used in America for age-related pediatric reference intervals for serum Ig levels, we noted differences in the reference values (5,7,8). Based on this observation, we conducted the present study to determine whether this variation resulted in differences in the detection of children with low Ig levels according to age. The clinical evaluation of the patients could not be performed in the study in the cases where Ig levels were found low.

Using the reference intervals determined by Tezcan et al., we found that 30.6% of the patients in our study were deficient in one or more Ig values. This was approximately 4 times greater than rates determined based on reference intervals reported by Aksu et al. (7.7%) and those in the *Nelson Textbook of Pediatrics* (6.8%). The prevalence of low Ig level was comparable when using the Aksu et al. and *Nelson Textbook *reference intervals.

Studies on serum Ig reference intervals have shown that these values vary depending on the methods used, the patient population, age, and sex (15–17). In addition, these reference intervals are also influenced by genetic, ethnic, and regional differences (18). The difference between the reference system determined by Tezcan et al. and the reference system in the *Nelson Textbook* may be attributable to the method used or to ethnic and regional differences.

In contrast, the two reference systems determined through studies conducted in our country both reflect values in the Turkish population. Therefore, the discrepancy between these two reference systems is less likely to be a result of ethnic and regional differences. These two systems were created using different analytical methods. Tezcan et al. used the turbidimetric method in their study, whereas Aksu et al. used the nephelometric method.

Both turbidimetry and nephelometry are based on measuring the scattering of radiation passed through a solution containing suspended particles (19). The turbidimetric method measures the intensity of the light that is transmitted through the solution to determine the light loss due to scattering by the suspended particles, whereas the nephelometric method measures the rays that are deflected by the suspended particles to a photocell positioned at a 90-degree angle to the incident axis (20). The IFCC and Laboratory Medicine’s Committee on Plasma Proteins recommend both immunonephelometry and immunoturbidimetry as reference methods for quantifying IgG, IgA, and IgM in serum or plasma (21). 

It is seen that there is even more difference between reference systems in patients under one year of age. Maturation of the immune system continues in first years of life. Therefore, we think it may be hard to determine the reference interval in this age group.

In summary, in this study we noted differences among three reference systems used for assessing age-related serum Ig levels in pediatric patients. These discrepancies can create confusion when evaluating the Ig results of patients. For example, a patient’s Ig value may be evaluated as low when using one reference system, while it would be considered normal according to another reference system.

This inconsistency between reference systems can lead to unnecessary advanced testing and a greater financial burden on the patient. Furthermore, the results of scientific studies can be reported very differently according to the reference values used.

In order to eliminate the disparity between the existing reference systems, new studies including larger numbers of healthy children and utilizing the same method should be conducted to determine more accurate age-related serum Ig reference intervals.
